# Neuronal threshold functions: Determining symptom onset in neurological disorders

**DOI:** 10.1016/j.pneurobio.2024.102673

**Published:** 2024-10-09

**Authors:** Luc Jordi, Ole Isacson

**Affiliations:** a Neuroregeneration Institute, McLean Hospital / Harvard Medical School, Belmont, MA 02478, USA; b Department of Neurology and Program in Neuroscience, Harvard Medical School, Boston, MA, USA

**Keywords:** Neurodegenerative Disorders, Parkinson’s Disease, Functional Thresholds, Multisystem Disorders, Compensatory Mechanisms

## Abstract

Synaptic networks determine brain function. Highly complex interconnected brain synaptic networks provide output even under fluctuating or pathological conditions. Relevant to the treatment of brain disorders, understanding the limitations of such functional networks becomes paramount. Here we use the example of Parkinson’s Disease (PD) as a system disorder, with PD symptomatology emerging only when the functional reserves of neurons, and their interconnected networks, are unable to facilitate effective compensatory mechanisms. We have denoted this the “threshold theory” to account for how PD symptoms develop in sequence. In this perspective, threshold functions are delineated in a quantitative, synaptic, and cellular network context. This provides a framework to discuss the development of specific symptoms. PD includes dysfunction and degeneration in many organ systems and both peripheral and central nervous system involvement. The threshold theory accounts for and explains the reasons why parallel gradually emerging pathologies in brain and peripheral systems generate specific symptoms only when functional thresholds are crossed, like tipping points. New and mounting evidence demonstrate that PD and related neurodegenerative diseases are multisystem disorders, which transcends the traditional brain-centric paradigm. We believe that representation of threshold functions will be helpful to develop new medicines and interventions that are specific for both pre- and post-symptomatic periods of neurodegenerative disorders.

## 1. Parkinson’s Disease is a system disorder with progressive neuronal degeneration both in the brain and the periphery, where symptoms occur when functional tipping points are reached

Parkinson’s Disease (PD) is a debilitating progressive neurodegenerative disease that affects more than 10 million people around the world. PD is increasingly recognized as a spectrum disorder characterized by remarkable heterogeneity in genetics, clinical presentation, and pathological progression (Wüllner et al., 2023). Most prominent motor symptoms include tremors, rigidity and bradykinesia which occur due to an underlying degeneration of the dopaminergic neurons in the substantia nigra pars compacta when the threshold of 70 % depletion of dopaminergic synapses is exceeded (Brownell et al., 1998; Engelender and Isacson, 2017; Kalia and Lang, 2015; Wullner et al., 1994). Non-motor associated symptoms such as autonomic failure, sleep disorders, sensory impairment and gastrointestinal dysfunction are due to a malfunction in the peripheral neurons and brain stem nuclei (Adler and Beach, 2016; Marras and Chaudhuri, 2016). Neurobehavioral changes like depression and anxiety are early-stage non-motor symptoms while in advanced stages disturbed executive functions, reduced vocal fluency and visuospatial abilities progressively become more prominent over the course of the disease (Gupta and Shukla, 2021; Maetzler et al., 2009; Reijnders et al., 2008). Dementia appears in late PD stages, similar in pathology to Lewy-body dementia and eventually affects around 80 % of PD patients in late stages (Hely et al., 2008).

The conventional characterization of PD primarily hinges on its central pathogenesis, with the primary focus on the dopaminergic neuron depletion in the substantia nigra (Fearnley and Lees, 1991). However recent evidence underscores a more expansive view of PD, highlighting it as a systemic disorder that impacts a broad spectrum of neural circuits spanning both the central (CNS) and peripheral nervous systems (PNS) supported by genetic cellular analyses that identified mutations in genes like LRRK2, SNCA, GBA and VPS35, which not only influence cellular behavior in the CNS but have implications for cellular processes in the PNS (Chang et al., 2017; Korecka et al., 2019; Ma et al., 2021; Nalls et al., 2014; Simon-Sanchez et al., 2009).

Central to this heterogenous and systemic disorder concept is the recognition of PD as a condition defined by highly specific functional thresholds. These function thresholds are not only inter-individually variable, giving rise to substantial differences in the clinical phenotype among patients (Wüllner et al., 2023), but also encompasses different cell populations, anatomic and neurological systems. Critical to understanding the progressive and sequential manifestation of disease symptoms is the profound age dependency observed across all major neurodegenerative disorders (Hou et al., 2019). In conditions such as PD, Alzheimer’s Disease (AD), and even Huntington’s Disease, individuals typically maintain a normally functioning central and peripheral nervous system until approximately the age of 45 for Huntington’s Disease (Duyao et al., 1993; Haque et al., 1997; Sharp and Ross, 1996), and with motor or dementia symptoms generally not emerging until their sixth or seventh decade of life for PD and AD respectively (Brownell et al., 1998; Pagano et al., 2016; Wullner et al., 1994). These observations underscore the conclusion that a series of complex interactions must occur, leading to functional deficits later in life. These deficits may result from cellular genetic alterations or analogous effects induced by biological aging through environmental factors, necessitating intricate interactions and compensatory phases before the eventual failure of brain and organ systems.

**Fig. 1.**
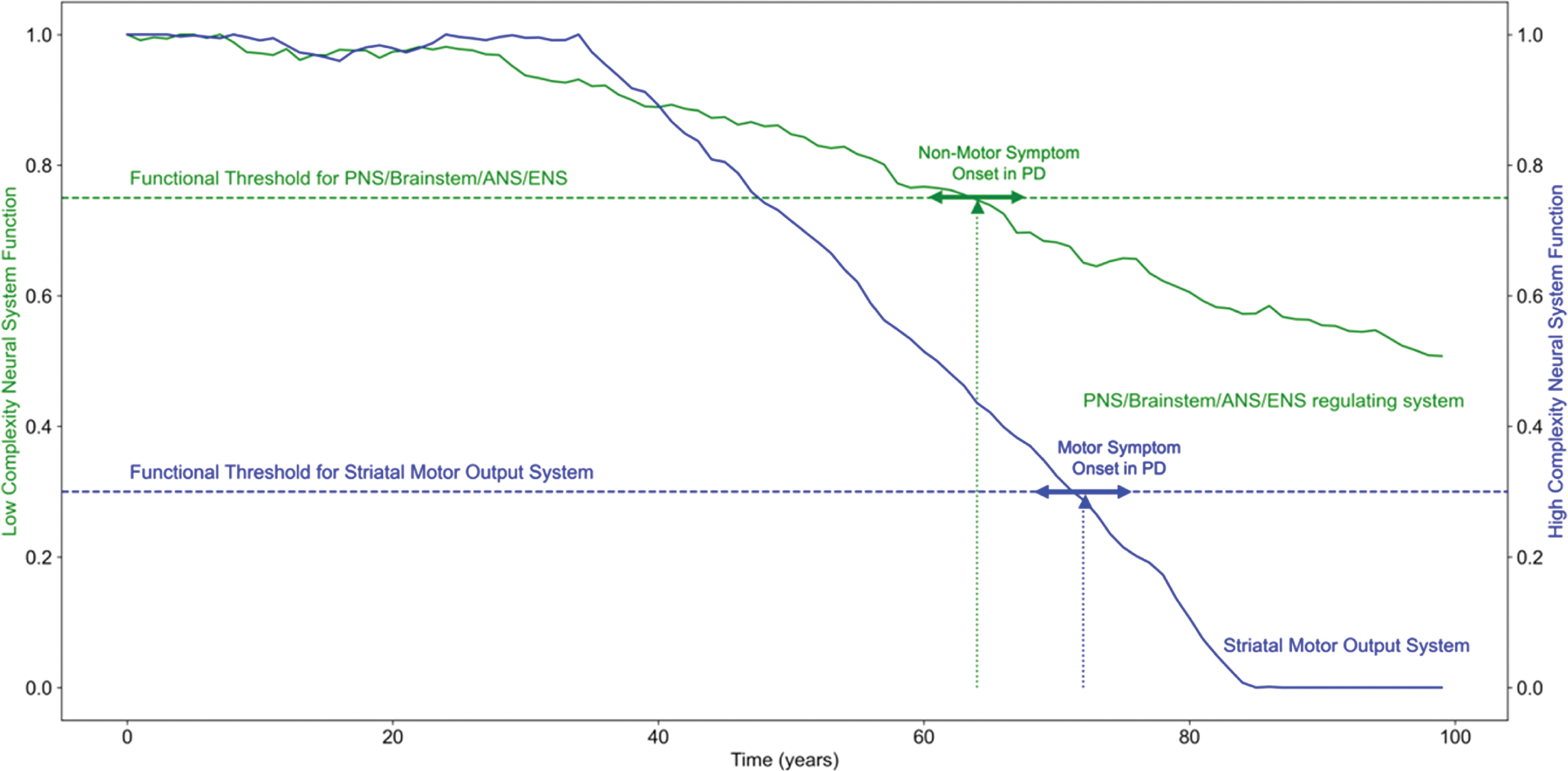
Functional resilience of complex motor integration in the caudate-putamen compared to less complex peripheral system failure. This model illustrates a conceptual representation of the framework proposed in the manuscript. The illustration was generated through multiple computational simulations using a range of predefined parameters including system-specific degeneration thresholds, varying levels of functional complexity, intrinsic system vulnerability, redundancy in neural circuits, temporal onset of degeneration, and the incorporation of stochastic noise. The functional threshold for striatal motor output system is based on our understanding of progressive decline in the midbrain to striatal connections of the dopaminergic neurons, and how they create symptoms at a certain point of dysfunction. The dopaminergic midbrain system decline in blue shows a pronounced loss of dopaminergic synapses but the measured threshold for parkinsonian motor symptoms only appears when 70 % of the synapses are degenerated as confirmed by PET-CFT, metabolic labeling and immunohistochemical assay. Brainstem-RAS, peripheral, autonomic and enteric nervous systems cellular pathologies can show early symptoms despite a relatively higher cellular resilience compared to the dopaminergic midbrain system. Consequently, a substantial 70 % loss of nigrostriatal dopaminergic synapses within the caudate-putamen system results in symptomatic manifestation much later compared to a mere 25 % pathological occurrence within the brainstem-RAS, peripheral, autonomic, and enteric nervous systems most likely due to different functional reserve which sheds light on the observed progression of disease symptoms. This conclusion is supported by certain non-motor symptoms, like constipation, hyposmia and REM sleep disorders, that can be discernible years before the emergence of motor symptoms in PD patients.

The concept of a threshold for the emergence of functional deficits is that symptoms appear only when compromised cellular systems become sufficiently dysfunctional (see Fig. 1). Such primary cellular dysfunction can be studied by lipid, mitochondrial or specific protein changes. One well known and extensively studied protein change in PD is the presence of aggregated α-synuclein in various nerve cells in PD and related disorders. As evidence of the systemic nature of this disease, cellular studies have shown α-synuclein aggregations not just in the CNS but also in enteric neurons, cardiac and pelvic plexus, epidermal nerve fibers, and the autonomic PNS (Beach et al., 2010; Doppler et al., 2014; Malek et al., 2014; Orimo et al., 2008; Rodríguez-Leyva et al., 2014). In PD patients with dysphagia, α-synuclein accumulations can be detected in the vagus nerve (pharyngeal motor and sensory branch) as well as in the glossopharyngeal and laryngeal nerve (Mu et al., 2013a; Mu et al., 2013b). The presence of α-synuclein elevation in peripheral tissues and the occurrence of peripheral neuropathy in PD underscores a more extensive involvement of the entire nervous system, advocating for a holistic view of its neurological impact (Chen et al., 2020; Corrà et al., 2023; Harms et al., 2021). Presence of α-synuclein is also evident in microglia and monocytes, indicating that the immune responses are widespread in CNS and peripheral regions (Klegeris et al., 2008; Lee et al., 2009). Additionally, peripheral changes in cells of the adaptive immunity like T-cells are observable (Harms et al., 2021). Fundamentally, non-motor manifestations of PD, such as constipation, hyposmia, and dream enactment of rapid eye movement sleep behavior disorder (RBD) provide clinical evidence of PD’s potential impact beyond the CNS, pointing towards a distributed pathology (Cersosimo et al., 2013; Doty et al., 1988; Edwards et al., 1991; Howell and Schenck, 2015; Pont-Sunyer et al., 2015; Sung et al., 2014) and supporting a view of PD as a multiregional systemic disease, influencing simultaneously different cellular systems across CNS and PNS.

When analyzing the heterogeneity of PD symptomatology, it becomes essential to consider the interplay of neuronal vulnerability, intrinsic adaptability, and compensation mechanisms at both localized and systemic levels. Neurons, depending on their location and function, possess varying vulnerability influenced by genetic, metabolic, connectivity or environmental factors (Sulzer, 2007; Surmeier et al., 2017). While some neuronal populations have inherent robustness and can adapt to pathophysiological changes through neuroplasticity, others might be more susceptible to degenerative changes (Aguila et al., 2021; Surmeier et al., 2017). However, beyond these inherent neuronal capabilities, the higher-level systemic compensation within neuronal groups plays a pivotal role, and perhaps the most important part of the threshold theory is to reimagine the operation of synaptic networks that maintain brain function and capacity, despite substantial synaptic and neuronal losses. Regional dysfunction of neurons depends on their individual and specific vulnerability but the symptomatic appearance results from the functional connection to and modulation from other neuronal groups and circuits (Engelender and Isacson, 2017). For these reasons, we consider functional thresholds as key to decipher the complex interplay among neurobiological systems that culminate in symptom manifestation.

## 2. Putting complex brain and nervous system function in perspective – introducing concepts of functional thresholds

Functional thresholds can be defined as inflection points when compensatory systems fail, and symptomatic deficits are apparent to the patient caregivers and medical professionals. Understanding these functional thresholds are integral to the intricate orchestration of neural systems and biochemical pathways that maintain cellular and systemic equilibrium.

The delineation of functional thresholds offers translational potential, especially when anchored in mathematically based multimodal network modeling. A threshold function-based model powered by AI-driven simulations, that considers multiple inputs such as age, cognitive scores and biomarker molecules, could identify when adaptive mechanisms will fail, shift to compensatory strategies, and lead to pathological symptoms. The symptomatic manifestations in PD can be perceived as the net outcome of the degree of functional connections and modulations of neurons within their corresponding networks. Dysfunctions in interconnected circuits lead to the clinical manifestations we observe (McGregor and Nelson, 2019). Moreover, to counterbalance these neuronal deficits, the brain exhibits adaptability, not only at a cellular level, for example, through changes in lipid metabolism and lysosomal function, altered membrane lipid composition, and functional changes in the exo- and endocytosis and vesicle transport pathways (Isacson et al., 2019), but also through higher-level systemic compensations to maintain functional and behavioral outputs. These compensatory mechanisms may manifest as functional overriding and selective activity adaptation of brain regions, activation of auxiliary neural circuits, functional redistribution within neural networks (Monchi et al., 2007; Rowe et al., 2002; Samuel et al., 1997; Wu and Hallett, 2013) and specific brain metabolic Parkinson related patterns (PRP) (Eidelberg et al., 1994).

To better account for complex versus more linear nervous system influences in PD and symptoms it generates in patients, we have proposed the threshold theory for PD to more effectively explain why symptoms can occur at different times in different systems in this disorder. In principle, the autonomic symptoms that include loss of smell, gastrointestinal dysfunction, loss of heart rate variation and orthostatic hypotension are all attributable to damage in sympathetic or olfactory nervous system damage due to the disease of either genetic and/or environmental origin (Adler and Beach, 2016; Arnao et al., 2020; Cersosimo et al., 2013; Doty et al., 1988; Hiorth et al., 2019; Jain and Goldstein, 2012; Marras and Chaudhuri, 2016; Sung et al., 2014). Such autonomic and peripheral systems are less complex and less integrated than the CNS brain regions. In the PD movement disorder, most of the motor dysfunction and the patient’s loss of initiation of movement, and to some extent rigidity, can be fully accounted for by a dysfunction of the caudate-putamen system, due to progressive loss of dopaminergic nigrostriatal synaptic input (Brownell et al., 1998; Wullner et al., 1994). The important insight from the scientific evidence and this theory is that the disease can advance more in CNS complex systems than in autonomic simpler systems before significant symptoms occur (Fig. 1). In other words, the threshold is higher in complex systems that are highly functionally integrated, with a high degree of biofeedback to maintain output functions (behaviors), than in more linear autonomic and peripheral systems, which, by definition, depend on automatic reflexive arcs. More specifically we and others have shown quantitatively that the primate and human cortical and caudate-putamen system can sustain a loss of up to 70 % of the specific nigrostriatal dopaminergic input before motor symptoms emerge (Brownell et al., 1998; Cooper et al., 2009; Hantraye et al., 1992; Wullner et al., 1994). The threshold for the autonomic, enteric and PNS is likely much lower and is predicted to occur at ranges of 25 % of dysfunctional synapses or less (Engelender and Isacson, 2017), due to the more linear construction and feedforward loops within such anatomic and organ systems (Fig. 1).

The significant diminution of the synaptic network function produces a loss of integrity of the set points for the emergence of symptoms, that are governed by large assemblies of synaptic networks. Notably, with elevated set points designated for such complex interactions, individual neurons may be dysfunctional, or not, before symptoms emerge. As evident by CNS labeling analyses, even disconnected neurons can maintain relatively unaltered synaptic morphology (Walker and McAllister, 1986; Wictorin et al., 1988). The threshold theory predicts that the dysfunctional assembly of a neuronal network produces the symptoms, rather than the isolated firing rates of individual neurons and their synapses. The functionality of specific neuronal assemblies and networks determines the extent of functional loss related to specific symptoms. When network activity declines significantly, it indicates a critical threshold has been reached, such as a total 60–70 % of loss of synaptic connections in movement disorders such as PD, as we have quantified specifically for nigrostriatal dopamine input to the caudate-putamen brain system (Brownell et al., 1998; Cooper et al., 2009; Hantraye et al., 1992; Wullner et al., 1994). This would also be true for functional deficits seen in other neurodegenerative diseases such as ALS, Lewy-Body dementia and AD. Many cognitive processes are executed through highly complex interactions and integration of information through parallel neural networks, rather than through signaling through a classic linear network representation of regional anatomic known pathways. It is therefore likely that the functional output in dementia disorders also follows the threshold theory, in that compensatory and adaptive mechanisms at a very complex level resets cognitive performance according to the best possible output (lack of symptoms). The threshold theory predicts that for cognitive disorders including dementias and AD, hippocampal and cortical memory functions will only result in symptoms at a cognitive level when the threshold and setpoint functions for such neuronal activity of the functional network reaches a high degree of disconnect between input and output systems. Through this lens, the threshold theory explains a multitude of neuronal system diseases, and other disorders stemming from synaptic dysfunction. The threshold theory predicts that the occurrence of symptoms in system disorders (including peripheral to brain) will vary with the complexity of the neuronal system. In scenarios involving CNS brain function with more complex interactions (Engelender and Isacson, 2017), a significant loss of individual synaptic activity, synaptic connectivity and elements is anticipated to precede symptoms onset. Indeed, it is not only a degeneration of synapses that is required for a loss or disturbance of complex networks and therefore brain function, but dysregulation of synaptic activity without any direct evidence of physical histological and obvious cellular dysfunction, can produce the same effect as synapse loss. The loss of synaptic control over an extended period and in regulated networks will eventually lead to behavioral output disturbances. This principle also extends to transient loss of synaptic function, such as in head trauma, stroke, and in neuro- inflammatory disorders. Such more acute events may also accelerate the transition to the threshold setpoint in susceptible brain regions and elsewhere, beyond which recovery is no longer possible.

## 3. From adaptation and compensation to loss of function in neurodegenerative diseases

The intricate neurobiological fabric of the brain is uniquely equipped with a remarkable ability to adapt and compensate in the face of progressing neural challenges.

In the early stages of such disturbances as sustained perturbations in synaptic or intrinsic properties alter neuronal spiking leading to a different single unit activation compared to their set-point (Mallet et al., 2006), as well as abnormal circuit synchronization and beta oscillations at the population level (Little and Brown, 2014; Moran et al., 2011), the brain harnesses on innate mechanisms of adaptation, predominantly regional and specific to the affected circuits or closely related local neuronal networks, which leads to an engagement of homeostatic mechanisms attempting to achieve a state of equilibrium (Marder and Goaillard, 2006; Turrigiano, 1999). Among these adaptive responses, up-regulation of dopamine (DA) transmission (Hornykiewicz, 1993; Pifl and Hornykiewicz, 2006) can be found as a possible early adaptive mechanism. Further reduced uptake of dopamine by the dopamine transporter (DAT) as shown by downregulation of DAT mRNA and PET scans, adjusting the availability of dopamine can be observed (Adams et al., 2005; Joyce et al., 1997; Lee et al., 2000). As we and others have shown in previous publications, those cellular adaptive homeostatic mechanisms are crucial for maintaining neural function under early conditions of stress (Chung et al., 2009; Chung et al., 2005).

Central to those functional brain compensatory mechanisms is the recruitment of alternative neural pathways. When the cardinal circuits, such as the nigrostriatal pathway, undergo neurodegeneration, the brain attempts to utilize redundant, alternative or auxiliary routes to sustain function, as seen by the increased activity of the pre-supplementary motor cortex (pre-SMA) in early PD during the performance of temporally self-initiated simple hand movements, increased dorsolateral prefrontal cortex (DLPFC) activity during walking in early stage PD patients or the increased activity in the cerebellum to potentially bypass faltering striatal circuits (Eckert et al., 2006; Ranchet et al., 2020; Wu and Hallett, 2013).

Progressing in tandem with the recruitment of auxiliary pathways is the adjusted connectivity or neuronal hyperconnectivity as a fundamental response to neurological disruption (Hillary and Grafman, 2017; Hillary et al., 2015). Extra-striatal basal ganglia (BG) modulation may be a major player in the compensation by modification of dopamine activation of output nuclei (Pifl et al., 1990) or by functional circuit adjustments (Galvan et al., 2014; Obeso et al., 2004). The adjusted connectivity is further supported by the presence of a shift in corticostriatal connectivity from severely affected striatal regions (posterior putamen) to less affected striatal regions (anterior putamen) in early-stage PD patients (Manza et al., 2016) or elevated functional connectivity (FC) between the MD/A thalamus and the anterior cingulate cortex in PD subjects, indicating a modulation of the basal ganglia-thalamocortical circuit aligning with their role in regulating cognitive task performance alongside the dorso-lateral prefrontal cortex (Fornito et al., 2004). Prolonged alteration in dopaminergic signaling further produce substantial reshaping of corticostriatal connections and their function (Surmeier et al., 2007). In particular, the role of the indirect pathway of movement control becomes pivotal when direct pathway functionality is compromised as it gets eventually upregulated as an effort to maintain control (Terkelsen et al., 2022) by the adaptive expression of late genes (Tsui and Isacson, 2011).

Metabolic PET studies have highlighted PD as a condition associated with abnormal activity across spatially distributed systems that influence motor and cognitive functions, through alterations in a set of functional brain networks, known as Parkinson related patterns (PRP) (Huang et al., 2007), indicating a potential role of additional brain regions in compensatory mechanisms. Lateral pre-motor cortex, SMA, DLPFC and parieto-occipital areas are characterized by hypometabolism in PD while hypermetabolism is present in the primary motor cortex, globus pallidus, thalamus and cerebellum (Eidelberg et al., 1994; Payoux et al., 2010). Several studies additionally suggest a globally increased excitability of the motor cortex at a systems level (Berardelli et al., 1996; Kojovic et al., 2015; Ni et al., 2013) and resting state fMRI independent component analysis (ICA) reveals functional connectivity alterations in intra -network connectivity, specifically the DMN (default mode network) and right frontoparietal network (RFPN) as well as inter-network connectivity alterations particularly implicating the ECN (Li et al., 2023). Further studies indicate that those neurocompensatory responses could be complemented by morphological changes like thickening and increased cortical surface area as well as increased gray matter volume in task-relevant brain structures (Biundo et al., 2013; Jubault et al., 2011; Shine et al., 2019; Wang et al., 2022). The canvas of compensatory mechanisms in PD also presents a potent portrait of the brain’s intrinsic reserve suggesting the concept of brain and motor reserve (MR) in PD patients, which may be linked with a more efficient and integrated utilization of functional brain networks (Chung et al., 2020; Shine et al., 2019). However, the capacity of the brain to maintain functional integrity against neurodegenerative challenges and stressors has limits. Evolving circuitry alterations and the ever-demanding compensatory endeavors will eventually converge on a functional tipping point. Beyond this threshold, function is lost and symptoms occur.

## 4. Low versus high complexity neural system function

When distinguishing between low and high complexity in neural system function, the degree of interconnection, and the potential for system compensation becomes central.

Pathophysiological alterations manifest as symptomatic occurrence depending on the functional reserve of each system affected, considering similar timing and severity degree. Low complexity neural systems allow less compensation and provide a narrower bandwidth of functionality, while high complexity neural systems allow for more compensation and therefore a higher functional threshold (Fig. 1).

The linear systems properties of neuronal pathways from the CNS to peripheral, autonomic, and enteric nervous systems highlight the absence of surplus connections and auxiliary pathways buffering their functionalities (Engelender and Isacson, 2017), attributing them a lower functional threshold for symptom emergence. In contrast, the complex striatal system circuitry governing movement initiation showcases diverse inputs to the striatal medium spiny neurons, which can offset deficits. For instance, the functional network of enteric neurons is considerably less intricate than the dopaminergic neuron circuitry governing movement initiation and control in the brain. Consequently, the enteric neuronal system tends to have a diminished functional reserve in comparison to the dopaminergic neuron circuitry. This discrepancy becomes evident when considering the extensive interconnectedness of human midbrain, striatal, pallidal, thalamic, and cortical nuclei (Alexander et al., 1986; DeLong and Wichmann, 2015). Such an elaborate network offers vast compensatory and redundancy mechanisms facilitating movement initiation. Thus, when subjected to analogous pathologies, the PNS, by virtue of its earlier functional threshold relative to the midbrain dopaminergic circuitry, manifests symptoms precedingly. This threshold function mechanism elucidates the early symptomatic progression in PD (Fig. 1).

Within this paradigm, the threshold theory offers a quantitative explanation for disease progression, underscoring the intrinsic connection between pathophysiological alterations translating into symptoms based on the functional reserve of each concurrently affected system and adeptly addressing the observed heterogeneity in PD symptomatology (Wüllner et al., 2023). Different neuronal systems, due to their unique regional and metabolic profiles, show varying vulnerability and resilience to PD pathologies by highlighting a systemic development of PD simultaneously in multiple foci across CNS and PNS (Pandya and Patani, 2021). Such variances are not only observed in the vulnerable A9 and more resilient A10 dopaminergic neurons (Chung et al., 2005) but are mirrored in other neuronal systems as well, emphasizing that vulnerability in PD is not a monolithic concept, but a spectrum influenced by multiple interconnected factors ranging from metabolic demands, variety of cellular stressors, the systems range of adaptation and to inflammation (Jagust, 2013; Surmeier et al., 2017).

The compensatory dynamics within systemic networks are critical for elucidating the resilience of neural systems to localized disturbances. Symptomatic manifestations occur only after the depletion of a system’s functional reserve, a phenomenon that is more pronounced in less complex neural architectures compared to highly interconnected and complex networks demonstrating enhanced robustness against perturbations, attributable to their extensive redundancy. This differential systems resilience accentuates the role of network complexity as a determinant of functional integrity in the face of localized impairments.

## 5. Understanding of biomarkers, neurological interventions and new medicines using the concept of functional thresholds

Understanding disease processes at the cellular, systemic, and integrated levels is crucial in determining how the underlying disease process manifests symptoms at varied rates. Such an understanding is essential for tailoring interventions that appropriately address the multifaceted and interconnected systems disease. The threshold theory proposes a nuanced view by examining the neurodegenerative disease as a cell biological vulnerability that impacts different systems at varying rates, yielding symptoms only when the threshold for compensation at a brain functional level is passed.

Specifically, the “α-synuclein spread theory” in PD potentially reveals a misconception that early symptoms are definitive indicators of pathology initiation at a certain anatomical location. Multiple reports contest the idea of pure protein ascension for pathology promotion and support that autonomous protein misfolding may emerge simultaneously in different regions proposed by our threshold theory (Attems and Jellinger, 2008; Engelender and Isacson, 2017; Hughes et al., 1993; Jellinger, 2008; Kalaitzakis et al., 2008; Kingsbury et al., 2010; Mori et al., 2002; Parkkinen et al., 2005; Parkkinen et al., 2008; Sorrentino et al., 2017; Espay and Lees, 2024). A likely consequence of solely targeting α-synuclein alterations through medical treatments is that will not be sufficient to significantly impact the underlying disease process and its functional thresholds. In fact, the now over 20 genes shown to influence the risk for PD are frequently upstream of α-synuclein expression or proteinopathy links (Billingsley et al., 2018; Chang et al., 2017; Nalls et al., 2014; Simon-Sanchez et al., 2009; Thomas et al., 2023), indicating that various biological mechanisms are interacting to generate a late stage pathology.

**Fig. 2.**
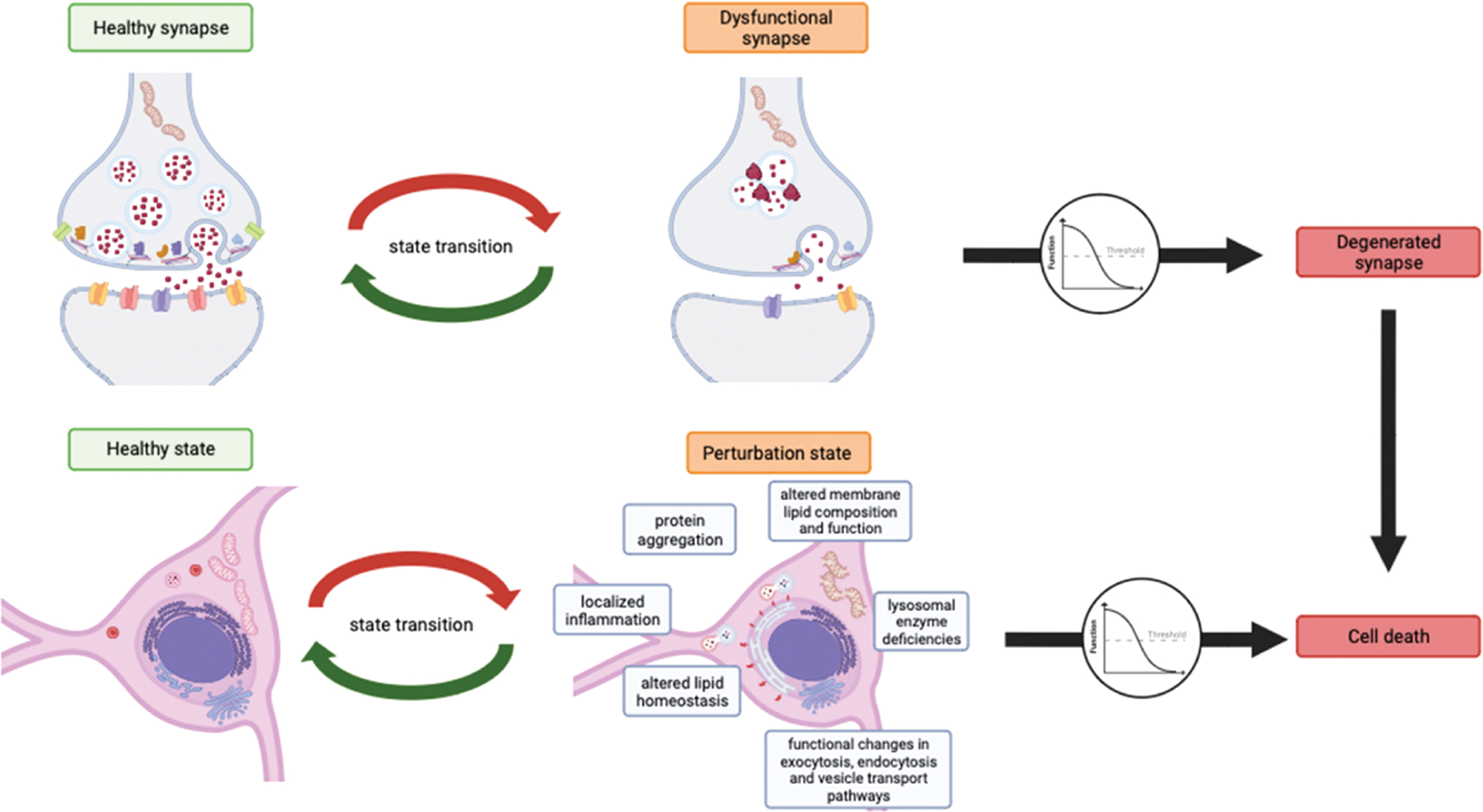
Cellular pathogenic mechanisms leading to substantial neuronal, glial and tissue response followed by synaptic degeneration over time in the context of PD. Neuronal changes in PD encapsulate a continuum that ranges from a healthy state through perturbation to eventual degeneration. Initially, neurons are in a state of equilibrium, characterized by balanced metabolic state and demand, lipid homeostasis and normal protein levels and distribution. This healthy state ensures optimal synaptic function as well as neuronal and systemic integrity. As neurons become subject to pathogenic stressors, they transition to a perturbation state. Here, disruption in lipid metabolism as well as further alterations like localized inflammation, functional changes in the exo- and endocytosis as well as the vesicle transport pathways, alongside lysosomal enzyme deficiencies, altered membrane lipid composition and function and protein aggregation begin to manifest. This perturbation state retains a potential for reversibility, contingent upon the neuron’s adaptive capacity and systemic support to resolve the perturbation as indicated by the bidirectional arrows. This transition function, however, is finite. Escalating stressor load and overwhelmed system compensation leads individual systems transitioning into a degenerative phase where system dysfunction becomes severe and irreversible.

A large window of treatment opportunity is in the pre-symptomatic phase of disease progression, where cells within synaptic networks engage in adaptation and compensation, striving to maintain network integration despite underlying pathological changes. Tackling neuronal stressors in this perturbation phase (see Fig. 2) holds the possibility to prevent pathological effects and regain a healthy homeostatic state. Shifting focus from the traditional medical concept of merely treating disease and symptoms to a more proactive approach of neuronal health enhancement can profoundly impact the disease course of PD. According to the threshold theory, by acting preemptively and addressing minor neural systems perturbations early on, one can positively impact the neuronal systems health trajectory and potentially prevent or delay further system distortion (Fig. 2), which is more relevant for developing impactful medicines.

To accomplish pre-symptomatic treatment, early biomarkers can serve as tools to intervene at diverse stages to mitigate or diminish the progression of the disease. Alterations of biomarkers in the blood, cerebrospinal fluid (CSF), brain, or peripheral systems, including the gastrointestinal tract, can be statistically linked with the onset of pre-symptomatic or the advancement to post-symptomatic disease states (Chahine et al., 2014; Nowak et al., 2022; Xiang et al., 2022; Yamashita et al., 2023). Moreover, the threshold theory predicts, effective future treatments will be multifaceted, addressing the disease comprehensively at a systemic level. Such interventions will likely encompass a combination of environmental, health to aging, and pharmacological strategies, customized to the specific stage of dysfunction, disease progression, and neuronal loss. The focus on the early phase highlights the importance of biomarkers research and development as this is the limiting factor for early detection and indication for potential treatment interventions pointing to a pivotal juncture where intervention can potentially prevent the crossing of critical functional thresholds. Therefore, to be able to restore a homeostatic state before the systems cross its specific threshold and moves from a perturbation state to an irreversible state of dysfunction (Fig. 2), early detection by robust biomarkers is essential.

In this context, there is a risk of an overreliance on genetic markers, which although insightful, may not provide the comprehensive and clinical information that is crucial for developing effective interventions and medication. This limitation of genetic information can also be illustrated by challenges by developing medication for Huntington’s disease, which, despite its near-absolute penetrance and well-understood genetic underpinnings since 1983 (Gusella et al., 1983), still lacks effective treatments (Tabrizi et al., 2022). Further, caution must also be exercised when addressing non-genetic biomarkers as signs of pathology, as they might represent compensatory processes. Indeed, using extensive patient brain tissue samples from stages 0 (no obvious pathology) to 4, in Huntington’s disease and also skin biopsy fibroblast cells, we showed that the biochemical deficits in ubiquitin proteolytic activities (proteosome), mitochondrial deficits (complex II) and BDNF neurotrophic activities closely correlate at the cellular level, independent of pathology (Seo et al., 2004). The most plausible explanation therefore for the cell vulnerability at the most neuronal cell death outcome (caudate-putamen striatal neurons) is loss of capacity to compensate for these obvious biochemical stressors linked to the genetic change (Seo et al., 2004). Consequently, biomarkers and biological understanding is necessary to learn how to mitigate many of these physiological changes in the interest of the patient (Seo et al., 2004). Huntington’s disease also fits the description of systemic changes, with disease mechanisms that interfere particularly in vulnerable brain systems. Similar to PD, Huntington’s disease reaches a stage where synaptic systems are most likely compromised to an extent that symptoms will occur beyond a compensatory adaptive level of function.

The emphasis on the early phase is equally important considering the pivotal role of aging as one of the key players in PD pathology progression (Pang et al., 2019; Reeve et al., 2014; Rodriguez et al., 2015). Aging acts as a feedforward mechanism in PD, by pushing brain organelle systems like lysosome and mitochondrial systems towards their limits in terms of functional thresholds (Chistiakov et al., 2014; Henrich et al., 2023; Nixon, 2020). These aging-related alterations set-up a system that becomes increasingly challenging to treat, necessitating interventions not only to be as early as possible, but also take the physiological state and trajectory of the perturbed system into account. With the system adaptation and compensation failing and eventually surpassing the functional threshold in different systems at different timepoints presents an opportunity for location-specific (CNS, peripheral systems) and systemic in-parallel interventions to restore function above the functional threshold and reduce progression by different agents tailored to the specific pathological characteristics of each system, pivoting in a nuanced and diversified holistic treatment strategy with focus on systemic interventions to influence several systems at the same time.

Functional thresholds in medicine and especially in PD should be understood as the aim to restore systems above a certain critical functional threshold and therefore going beyond a dose-response effect. Highly integrated systems as the intricate network systems in the brain eventually do not follow a linear dose-response curve but are better understood as integrated feedback interactions with functional thresholds for subsystems and overarching integrated systems. As an example of how interventions depend on the functional stage, the restoration of dopaminergic synapses using neuronal cell replacement therapy to move the systems functionality above the functional threshold, shows substantial promise in yielding positive outcomes by providing sustained benefits for up to 15–20 years as it increases dopamine levels and restores synapses, allowing autoregulation of synaptic dopamine release between the newly grafted cells and the host brain and therefore restores the functionality by moving the system above the functional threshold (Osborn et al., 2020; Strecker et al., 1987; Tsui and Isacson, 2011; Zetterstrom et al., 1986). Similarly, alternative interventions like deep-brain stimulation (DBS) operate and modulate at a functional level mitigating dysfunctional neural systems in disease states, thus offering an adjustable, patient-specific tailored method for functional restoration due to network modulation (Lozano et al., 2019).

The threshold theory discussed here focuses on the loss of functionality in neuronal networks due to crossing brain functional thresholds, instead of a simple linear theory based on observations of pathological tissue progression of disease. The threshold theory approaches neurodegenerative diseases as systems disorder, which encompass cellular, systemic, and integrated levels of biological organization. We believe the threshold theory will be helpful for future medical interventions in diseases such as PD and related disorders, by providing insights to patient specific, time- and system-tailored treatments.

## Data Availability

No data was used for the research described in the article.
